# Oncoprotein DEK as a tissue and urinary biomarker for bladder cancer

**DOI:** 10.1186/1471-2407-11-234

**Published:** 2011-06-10

**Authors:** Antara Datta, Martin E Adelson, Yakov Mogilevkin, Eli Mordechai, Abraham A Sidi, Jason P Trama

**Affiliations:** 1 Oncoveda, Tumor Biology Center, Medical Diagnostic Laboratories, A Division of Genesis Biotechnology Group, Hamilton, New Jersey, USA; 2 Department of Urology, The E. Wolfson Medical Center, Holon, Israel and the Sackler Faculty of Medicine, Tel-Aviv University, Israel

## Abstract

**Background:**

Bladder cancer is a significant healthcare problem in the United States of America with a high recurrence rate. Early detection of bladder cancer is essential for removing the tumor with preservation of the bladder, avoiding metastasis and hence improving prognosis and long-term survival. The objective of this study was to analyze the presence of DEK protein in voided urine of bladder cancer patients as a urine-based bladder cancer diagnostic test.

**Methods:**

We examined the expression of DEK protein by western blot in 38 paired transitional cell carcinoma (TCC) bladder tumor tissues and adjacent normal tissue. The presence of DEK protein in voided urine was analyzed by western blot in 42 urine samples collected from patients with active TCC, other malignant urogenital disease and healthy individuals.

**Results:**

The DEK protein is expressed in 33 of 38 bladder tumor tissues with no expression in adjacent normal tissue. Based on our sample size, DEK protein is expressed in 100% of tumors of low malignant potential, 92% of tumors of low grade and in 71% of tumors of high grade. Next, we analyzed 42 urine samples from patients with active TCC, other malignant urogenital disease, non-malignant urogenital disease and healthy individuals for DEK protein expression by western blot analysis. We are the first to show that the DEK protein is present in the urine of bladder cancer patients. Approximately 84% of TCC patient urine specimens were positive for urine DEK.

**Conclusion:**

Based on our pilot study of 38 bladder tumor tissue and 42 urine samples from patients with active TCC, other malignant urogenital disease, non-malignant urogenital disease and healthy individuals; DEK protein is expressed in bladder tumor tissue and voided urine of bladder cancer patients. The presence of DEK protein in voided urine is potentially a suitable biomarker for bladder cancer and that the screening for the presence of DEK protein in urine can be explored as a noninvasive diagnostic test for bladder cancer.

## Background

Bladder cancer is the sixth most prevalent malignancy in the United States of America, with an expected 70,530 newly diagnosed cases in 2010, and 14,680 deaths [1]. More than 90% of bladder cancers are transitional cell carcinomas (TCC), 5% are squamous cell carcinomas, and less than 2% are adenocarcinomas. Urothelial tumors are classified into four categories: papilloma, papillary urothelial neoplasm of low malignant potential, low grade carcinoma, and high grade carcinoma [2]. Of all newly diagnosed cases of transitional cell carcinomas, about 75% present as superficial tumors. Of those superficial tumors, 50 to 70% will recur and roughly 10 to 20% will progress to aggressive invasive disease [3]. Patients are therefore kept under surveillance for early detection of recurrences. Early and accurate detection of bladder cancer will allow for effective treatment of bladder cancer patients, hence improving prognosis and long-term survival.

Cystoscopy, the current "gold standard" clinical procedure to detect bladder cancer is an invasive, unpleasant and expensive method with poor patient compliance. Urine-based detection of bladder cancer biomarkers aims to replace or reduce the use of cystoscopy for diagnosis and surveillance of bladder cancer. However, urine cytology, and many of the currently developed FDA approved urine biomarkers including detection of chromosomal aneuploidy and deletion using fluorescence *in situ *hybridization (UroVysion^®^) have limited sensitivity for detection of low stage and grade tumors that form the main group that recur [4,5]. Therefore, there is a need for more sensitive urinary biomarkers, exemplified by recent data regarding epigenetic [6] and protein biomarkers[7], that can be implemented in molecular diagnostic laboratories

The DEK protein was initially identified as a fusion protein with CAN nucleoporin in a subtype of acute myeloid leukemia involving the t(6;9) chromosomal translocation[8]. The oncogene DEK is overexpressed in several malignancies including melanoma, hepatocellular carcinoma, glioblastoma, retinoblastoma, and bladder cancer [9-11]. Furthermore, autoantibodies to DEK have been detected in juvenile rheumatoid arthritis [12], systemic lupus erythematosus and sarcoidosis [13]. Proto-oncogenic roles of DEK includes the ability to inhibit p53 mediated apoptosis [14], cooperate with the viral oncogene E6 and E7 to overcome senescence [15] and promote epithelial transformation *in vitro *and *in vivo *when overexpressed [16].

A region of genomic gain on chromosome 6p22 that has been detected in high grade bladder cancer tumors contains the DEK gene [9]. cDNA microarray analysis of bladder tumor tissues found DEK as one of the genes significantly overexpressed in early stage bladder tumors [17]. However, there is no published literature to support increased DEK protein levels in bladder tumors and in the urine of bladder cancer patients.

Here we show that oncoprotein DEK can be used as a biomarker for detection of bladder cancer using patient urine samples. We evaluated DEK protein levels in bladder tumor tissues and found high levels of DEK protein in tumor tissue with no expression in adjacent normal bladder tissue. Next, we determined if the DEK protein is present in urine of bladder cancer patients. We are the first to show that DEK protein can be detected in the urine of bladder cancer patients by western blot analysis using a commercially available polyclonal DEK antibody.

## Methods

### Tissue specimen collection and protein isolation

Bladder tumor tissue and corresponding noncancerous bladder tissues were obtained from consented patients under Israeli Ministry of Health (Protocol no. 902008-0588) and Institutional Review Board (Protocol no.1091) approved protocols from Wolfson Medical Center, Holon, Israel.

All patients had positive findings on initial diagnosis by cystoscopy. Tissue samples were immediately frozen after removal and stored at - 80°C. A section of the tumor tissue was sent for histologic diagnosis to determine tumor grade and stage and results were sent along with the samples. The patients' background and clinical data are summarized in Table 1.

**Table 1:** Patient population and characteristics

Bladder Cancer (TCC)	Tissue	Urine
Total number	38	19
Mean age (range)	76 (53-87) years	74 (53-85)
Gender		
Male	32	17
Female	6	2
Grade		
Low malignant potential	10	3
Low grade	14	8
High grade	14	8
Stage		
Superficial (pTa)	18	10
Invasive (≥ pT1)	20	9
**Prostate Cancer (CaP)**		
Total number	-	7
Mean age (range)	-	60 (55-71) years
**Renal Cell Carcinoma (RCC)**		
Total number	-	4
Mean age (range)	-	61(53-72) years
**Healthy**		
Total number	-	6
Mean age (range)	-	37(32-49) years
**Suspected TCC and pathological diagnosis**		
Total number	6	6
Cystitis/ Chronic Inflammation	3	3
Squamous metaplasia and BPH	1	-
No tumor	2	1
Benign renal tumor	-	2

Cold cut frozen tissue samples were homogenized in RIPA buffer (150 mM NaCl, 0.01 M sodium pyrophosphate, 10 mM EDTA, 10 mM sodium fluoride, 50 mM Tris pH 8.8, 0.1% SDS, 12.8 mM deoxycholic acid, 10% glycerol, 1% NP-40) using a mortar and pestle. Sixty (60) ug of tissue extracts were resolved on a 10% SDS-PAGE gel and analyzed for DEK expression by western blot using a monoclonal antibody (BD Bioscience, San Diego, CA).

### Urine specimen collection and protein isolation

Under Israeli Ministry of Health (Protocol no. 902008-0588) and Institutional Review Board (Protocol no.1091) approved protocols from Wolfson Medical Center, Holon, Israel, voided urine from patients with bladder cancer (i.e., TCC), prostate cancer (i.e., CAP) and renal cancer (i.e., RCC) were collected on the day of surgery to remove the diagnosed tumor. The patients' background and clinical data are summarized in. Urine samples (~20-50 ml aliquots) were stored in the presence of a protease inhibitor tablet (Roche, Indianapolis, IN) and maintained at -80°C.

Proteins from 20 ml of urine were precipitated with two volumes of ice-cold acetone and incubated at -20°C for 1 hour. Post centrifugation, the acetone precipitated protein pellet was suspended in 2 ml of sucrose buffer (10 mM triethanolamine and 250 mM sucrose). Four hundred (400) μl of the resuspended urine protein was further concentrated using a 3K Microcon^® ^filter and was subjected to western blot analysis using a polyclonal DEK antibody (Bethyl laboratories, Montgomery, TX).

### Cell Lines

Human bladder cancer cell lines T-24 and RT-4 were maintained in McCoy's 5A medium supplemented with 10% fetal bovine serum (FBS) and human bladder cancer cell lines 5637 and TCCSUP were maintained in RPMI supplemented with 10% FBS. SV-40 transformed human bladder urothelial cell line (i.e., UroTSA cell line) was maintained in DMEM medium supplemented with 10% FBS. Human bladder epithelium progenitor cell line (i.e., HBEP) (CELL N TEC^®^, Stauffacherstrasse, Bern, Switzerland) was maintained in CnT-58 medium. Differentiated epithelial cells were maintained in accordance with the manufacturer's protocol (CELL N TEC^®^, Stauffacherstrasse, Bern, Switzerland). All cell lines were maintained at 37°C in 5% CO_2_.

### Whole Cell Lysates from Cell Lines

Prior to cell lysis, cultured cells were washed with 10 ml of cold PBS. Cells were lysed using 1 ml of RIPA buffer supplemented with protease inhibitors (Roche, Indianapolis, IN) at a concentration of 1 μg/μl. Lysed cells were scraped and transferred to a 1.5 ml centrifuge tube and centrifuged at 14,000 rpm for 10 minutes to collect supernatant (i.e., whole cell lysates).

### DEK Knockdown and DEK Overexpressing Cell Line

A lentiviral vector containing the DEK shRNA (pGIPZ-DEK shRNA) (cat. no. RHS4430-99137795) was purchased from Open Biosystems (ThermoScientific, Huntsville, AL). To prepare DEK-shRNA lentivirus, 5 × 10^5 ^293FT cells were first transfected with 10 μg of pGIPZ-DEK shRNA and 5 μg of the packaging vectors (i.e., pCMVΔR8.2 and pHCMV-G) and grown at 37°C in 5% CO_2_. Supernatants of the transfected cells (containing lentivirus particles) were collected at 24 and 48 hours post-transfection. To obtain the DEK knockdown 5637 cell line, 5637 cells were transduced with DEK shRNA lentiviral particles and selected using 2.5 μg/μl puromycin (Sigma-Aldrich, St. Louis, MO).

DEK cDNA (NCBI Accession No. NM_003472.3) was cloned into a pLenti GATEWAY^® ^lentiviral vector and packaged as virus using ViraPower™ BSD Packaging Mix (Invitrogen, Carlsbad, CA). Five (5) ml of the virus-containing filtrate was spread onto a 10 mm tissue culture dish containing confluent 5637 cells. Cells were selected for DEK-V5 expression at 48 hours using 10 μg/μl blasticidin (Invitrogen, Carlsbad, CA).

## Results

### DEK expression in bladder cancer cell lines

**Figure 1:**
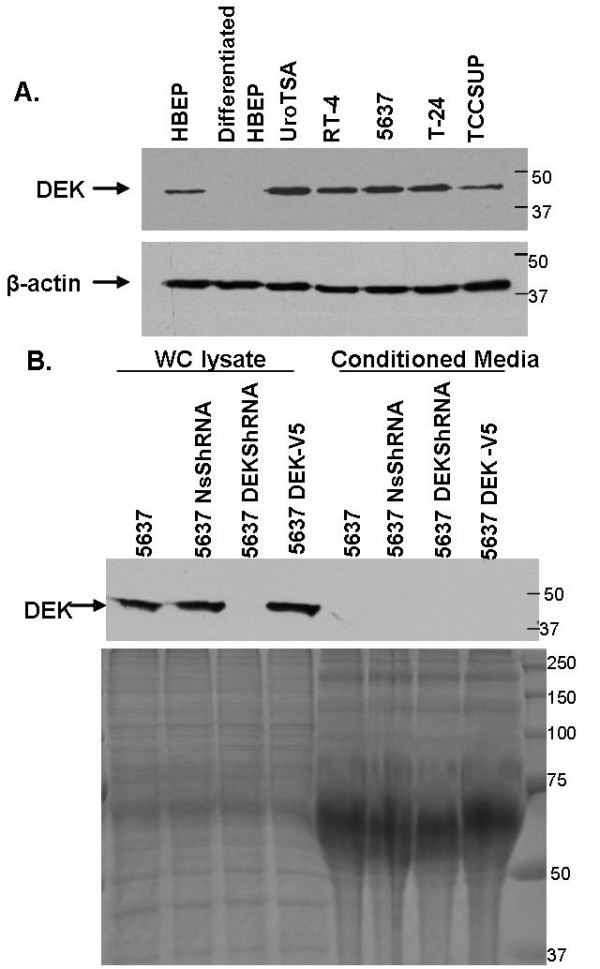
**DEK expression in cell lines**. **A**. Whole cell lysates were prepared from bladder cancer cell lines (RT-4, 5637, T-24 and TCCSUP), Urothelial cell line (UroTSA) and undifferentiated and differentiated progenitor bladder epithelial cell lines. DEK protein was evaluated by western blot analysis using a DEK monoclonal antibody (BD Bioscience). β-actin was used as a loading control. **B**. Expression of DEK protein in whole cell (WC) lysate and respective conditioned media from 5637, 5637 expressing nonspecific shRNA (Ns shRNA), DEK shRNA or overexpressing DEK with a V5 tag (DEK-V5) by western blot assay using a monoclonal DEK antibody. Bottom panel represents a Coomassie stained gel to show that equal amounts of proteins were loaded on the gel.

Previous studies based on tissue microarray data indicated that DEK mRNA is overexpressed in bladder tumors [17]. To test if high levels of DEK protein are present in bladder cancer cell lines, we prepared whole cell lysates using RIPA buffer from bladder cancer cell lines RT-4, 5637, T-24 and TCCPSUP derived from increasing grades of bladder cancer. We also prepared lysates from UroTSA cells which are normal bladder epithelial cells transformed with SV-40 T-antigen. Western blot analysis was performed using a DEK monoclonal antibody (BD Biosciences). Results from western blot analysis indicate that DEK is expressed in all bladder cancer cell lines as well as in normal bladder epithelial cell line UroTSA. (Figure 1A). To test if DEK is present in only proliferating cells, we used human bladder epithelial progenitor cell line (HBEP) that proliferate in growth media and induced them to a non-cycling differentiated state with 1 mM calcium chloride. Lysates were prepared from HBEP and differentiated HBEP and analyzed by western blot using the DEK monoclonal antibody. Results indicate that DEK protein is lost upon differentiation of primary cells (Figure 1A).

Previous studies have demonstrated that patients with Juvenile Rheumatoid Arthritis (JRA), have auto-antibodies against DEK protein [12] and DEK protein is found to be secreted in the synovial fluid of JRA patients [18]. To test if bladder cancer cells also secrete DEK protein, we analyzed conditioned media obtained from bladder cancer cell line 5637. Furthermore, we established two stable cell lines: one that overexpressed DEK protein with a V5 tag in the 5637 cell line (5637DEK-V5) and the other expressing DEK shRNA to knockdown DEK expression (5637 DEK shRNA). Conditioned media from these two cell lines were also analyzed for DEK expression.

Results show that DEK is absent in the conditioned media of bladder cancer cell line 5637 indicating that DEK may not be secreted by bladder cancer cells (Figure 1B). Furthermore, even overexpression of DEK protein in 5637 cell line (5637 DEK-V5) did not result in secretion of DEK protein in the conditioned media (Figure 1B).

### DEK expression in bladder tumor tissue

Several reports indicate that DEK is associated with bladder tumor tissue, which includes mRNA overexpression studies by microarray [10,17] and amplification of 6p22.3 region of chromosome, wherein the DEK gene is located, in 25% of advanced stage bladder cancer [9]. We wanted to test if DEK protein is expressed in bladder tumor tissue samples as compared to adjacent normal tissue. We obtained 38 TCC tissue samples, confirmed by pathology from Wolfson Medical Center, Holon, Israel.

**Figure 2:**
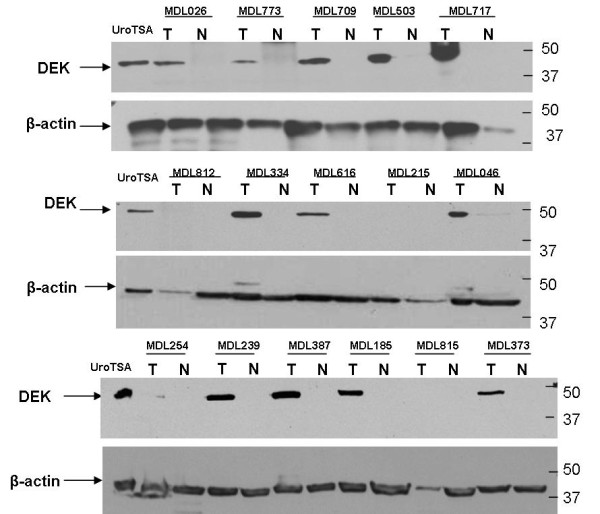
**DEK expression in bladder tumor tissue**. Tissue lysates from bladder tumor tissue (T) or paired adjacent normal tissue (N) were analyzed by western blot using DEK monoclonal antibody (BD Biosciences). An established bladder SV-40 transformed urothelial cell line (UroTSA) lysate was used as a positive control. β-actin was used as a loading control. Sample MDL815 represents a biopsy confirmed inflamed tissue from bladder (no tumor present). Three representative western blots are shown.

Whole cell lysates were prepared from tumor tissue and adjacent normal tissue samples and analyzed by western blot using a DEK monoclonal antibody (BD Biosciences). Results show that 33 out of 38 (86%) TCC tumor tissues of both low and high grade were positive for the presence of DEK protein with no detectable levels of DEK protein in the adjacent normal tissues (Figure 2). DEK protein is present in 100% of low malignant potential (LMP) TCC tissue and 93% in low grade TCC whereas only 71% of high grade tumor tissues were positive for DEK expression (Table 2). We were unable to detect DEK in four high grade TCC and one low grade TCC.

**Table 2:** DEK tissue expression in different grades of TCC

	TCC				
			
	Tumor Grade				
			
DEK WB	LMP	Low	High	NAT	NMC
**Positive**	10	13	10	0	2

**Negative**	0	1	4	38	3

DEK protein expression by western Blot (WB) in transitional cell carcinoma (TCC) tissue, Normal adjacent tissue (NAT) and tissue from nonmalignant conditions (NMC). LMP represents low malignant potential grade.

In our study we had five (5) samples from patients who were suspected of bladder tumor by cystoscopy or by the presence of gross hematuria but upon pathological analysis of surgically removed suspected areas, the tissues were found negative for tumor. However, by western blot analysis of another segment of the tissue we found DEK protein to be present in 2 out of the 5 samples. We cannot rule out the possibility that DEK protein may be present in the bladder tissue of patients with cystitis. Overall, our data further strengthens the association of DEK with bladder cancer and as a potential marker of bladder cancer.

### DEK expression in urine

**Figure 3:**
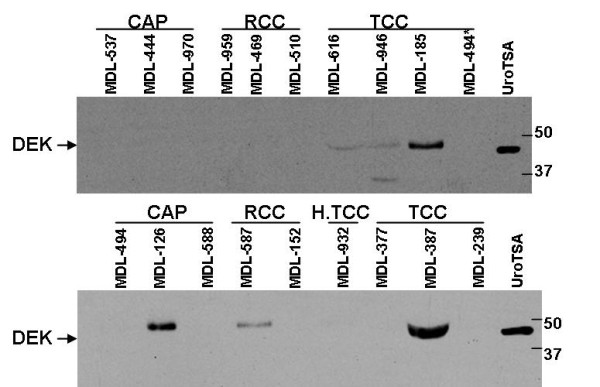
**DEK expression in urine**. Thirty (30) ul of acetone precipitated urine protein lysates were analyzed by western blot using a polyclonal DEK antibody (Bethyl Laboratories). An established bladder SV-40 transformed urothelial cell line (UroTSA) lysate was used as a positive control. CAP indicates prostate cancer, RCC indicates renal cell carcinoma, H.TCC indicates history of transitional cell carcinoma and TCC indicates transitional cell carcinoma of the bladder. Voided urine sample, MDL 494* was collected from a suspected TCC patient but pathology report was inconclusive. Two representative western blots are shown.

Our data indicates that DEK protein is present only in bladder tumor tissue. As urine contains both cells exfoliated from normal and cancerous urothelium as well as proteins from either secretion or cell lysis, we wanted to test if DEK protein can be detected in the urine of patients with TCC. Forty-two (42) voided urine samples from patients with active bladder cancer (TCC), prostate cancer (CAP), renal cancer (RCC), healthy subjects and urine from patients with suspected TCC which were found positive for nonmalignant conditions (NMC) by pathology, were analyzed for the presence of DEK protein. Results show that DEK protein is detected in 16 out of 19 urine samples of bladder cancer patients (Figure 3). The DEK protein is detected in the urine of patients with both low and high grade TCC. Based on the indicated sample size, the sensitivity and specificity of detecting DEK in the urine of bladder cancer patients were 84% and 83% respectively with a positive predictive value of 80% and negative predictive value of 86% (Table 3). However, we also detected DEK protein in the urine of some prostate and renal cancer patients indicating that DEK protein may be overexpressed in some prostate and renal cancer tumors.

**Table 3:** Detection of DEK protein in voided urine

	TCC			CAP		RCC					
			
	Tumor grade			Gleason's Score		Clear Cell Type: Fuhrman's Grade			Papillary Cell Type: Fuhrman's Grade		
			
DEK WB	LMP	Low	High	6 (3+3)	7 (3+4)	1	2	3	Unknown	Healthy	NMC
**Positive**	3	6	7	1	0	0	0	1	1	0	1

**Negative**	0	2	1	4	2	1	1	0	0	6	5

The presence of DEK protein in voided urine by western Blot (WB) in transitional cell carcinoma (TCC), Prostate cancer (CAP), renal cell carcinoma (RCC) and nonmalignant conditions (NMC). LMP represents low malignant potential grade.

Upon comparison of DEK protein levels in tissue and in the voided urine from TCC patients, we found 12 out of 18 samples were consistent for DEK expression in tumor tissue and voided urine. Six (6) samples had DEK protein expression in either tissue or voided urine. However, the two inflammation samples that were positive for DEK in tissue were found to be negative for the presence of DEK protein in voided urine. (Table 4)

**Table 4:** Comparison of DEK protein expression in tissue sample and voided urine from patients with TCC

Sample ID	Clinical Data	Urine	Tissue (T/N)
**MDL196**	TCC Ta low malignant potential	positive	+/-

**MDL185**	TCC Ta low malignant potential	positive	+/-

**MDL492**	TCC Ta low malignant potential (I)	positive	+/-

**MDL616**	TCC Ta low grade (I-II)	positive	+/-

**MDL254**	TCC Ta low grade (I)	positive	+/-

**MDL503**	TCC Ta low grade(II)	positive	+/-

**MDL709**	TCC Ta low grade (I)	positive	+/-

**MDL812**	TCC Ta low grade (I) with squamous differentiation	positive	-/-

**MDL377**	TCC Ta low grade (II)	negative	+/-

**MDL239**	TCC T1 low grade	negative	+/-

**MDL387**	TCC T1 high grade with pappilary+ solid pattern	positive	+/-

**MDL946**	TCC T1 high grade(III) + CIS with solid pattern	positive	+/-

**MDL026**	TCC T1 high grade	positive	+/-

**MDL046**	TCC T2 high grade + CIS	positive	+/-

**MDL215**	TCC T2 high grade	positive	-/-

**MDL911**	TCC T2 high grade (III)	positive	-/-

**MDL773**	TCC T2 high grade (III)& Papillary RCC type1	negative	+/-

**MDL717**	TCC T2 high grade (III)	positive	+/-

**MDL300**	Cystitis	negative	+/-

**MDL612**	Chronic Inflammation	negative	+/-

T: tumor tissue

N: normal adjacent tissue

Grey indicates the discrepancy in results between urine DEK expression and tissue DEK.

## Discussion

As bladder cancer has the highest recurrence rate as compared to any other malignancies, diagnostic and disease monitoring tools that can provide high specificity and sensitivity would be of great interest in urological oncology providing enormous benefit to patients, particularly if the specimens could be obtained noninvasively.

The overall goal of this study was to determine if the expression of the DEK oncoprotein might serve as a biomarker for the detection of bladder cancer. Several studies indicated that DEK is overexpressed in bladder tumors. One of the most consistently amplified regions in advanced bladder cancers is located at 6p22.3 [19]. Oncogene DEK is located within this region. Furthermore, using bladder tumor cDNA microarrays, DEK mRNA was significantly up regulated in bladder cancer [10,17]. Based on these studies, one would predict that DEK protein might be associated with the development of bladder cancer.

In this study, we have identified DEK as a potential urinary and tissue biomarker for TCC of the bladder of both low and high grade, stage and progression. Eighty-six percent (86%) of bladder tumor tissues were found to be positive for DEK protein expression. Based on our data obtained from a relatively small sample size of 38 tumor samples, DEK protein is present in most low grade tumor tissue as compared to high grade.

We are the first to show that DEK protein is present in the voided urine of patients with TCC of both low and high grade. Based on our study, the presence of DEK protein is largely specific for bladder cancer with a few cases wherein DEK protein is present in renal and prostate cancer. This study is limited to detection of DEK protein in the urine of diagnosed TCC patients with first incidence or recurrence of bladder cancer and not in high risk individuals. Furthermore, we have a limited number of healthy individuals that are not age matched to the TCC and other malignant urogenital disease group.

We acknowledge that the presented data is based on a small sample size. However, based on our pilot study, oncoprotein DEK is a promising biomarker of bladder cancer. Furthermore, the presence of DEK protein in the urine of bladder cancer patients, makes the detection of DEK protein in the urine of patients as an attractive diagnostic for bladder cancer. We are encouraged to further analyze DEK as a biomarker of bladder cancer using larger number of patient samples that will allow us to further strengthen the presence of DEK protein in the urine of bladder cancer patients as a biomarker for bladder cancer. We analyzed a small number of prostate and renal cancer sample and we detected DEK in 16% and 50% of prostate and renal cancer respectively. We cannot rule out the possibility that DEK may be present in renal cancer. Therefore, we propose to analyze larger number of urine samples obtained from prostate and renal cancer patients to address this concern. We are in the process of initiating a prospective study involving three different centers, in which we will test the presence of urine DEK in suspected TCC patients, age matched healthy individuals, other malignant and nonmalignant urogenital disease patients and confirmed TCC cases.

As western blot analysis may not be the choice method for diagnostics, we are currently in the process of developing a DEK ELISA for sensitive and specific detection of DEK protein in voided urine sample. For DEK to be adopted into clinical use, DEK must significantly improve the predictive ability of current biomarkers. We will be comparing our assay with the FISH based test UroVysion^® ^and other urine based biomarkers like NMP22. We cannot rule out the possibility that DEK alone may not be as accurate as cystoscopy procedure. However, it is possible that multiplexing of DEK with other urinary biomarker may be as predictive as cystoscopy. Nonetheless, detection of DEK protein in voided urine samples alone or in combination with other biomarkers, if successfully validated, will provide a sensitive and noninvasive method of testing for bladder cancer and subsequent recurrences.

## Conclusion

This pilot study demonstrated that DEK protein is over-expressed in bladder tumor tissue but not in adjacent normal tissue. Furthermore, the DEK protein is present in the voided urine of patients with bladder cancer. The DEK protein is present in the voided urine of patients with low and high grade bladder cancer. These results indicate that the detection of the DEK protein in the voided urine may potentially be used as a diagnostic test for detection and surveillance of bladder cancer.

## Competing interests

Financial competing interests: The authors AD, JPT, MEA and EM are employees of Medical Diagnostic Laboratories, a division of Genesis Biotechnology Group, Hamilton, New Jersey, United States of America.

The research presented in this manuscript and article-processing charge for this manuscript is funded by Medical Diagnostic Laboratories.

The authors AD and JPT are named inventors on a patent application which includes data presented in this manuscript.

## Authors' contributions

All authors read and approved the final manuscript. AD was responsible for study design, experimental job, interpretation of the results and writing the manuscript. JPT contributed towards the conception and design of the study, interpretation of the results and critically reviewed and edited the manuscript. YM was responsible for data collection. MEA, EM, and AAS were responsible for data analysis and interpretation of the study.

## Pre-publication history

The pre-publication history for this paper can be accessed here:

http://www.biomedcentral.com/1471-2407/11/234/prepub
